# Neutrophil to Lymphocyte Ratio Is Increased and Associated With Left Ventricular Diastolic Function in Newly Diagnosed Essential Hypertension Children

**DOI:** 10.3389/fped.2021.576005

**Published:** 2021-05-19

**Authors:** Miao Hou, Lei Cao, Yueyue Ding, Ye Chen, Bo Wang, Jie Shen, Wanping Zhou, Jie Huang, Qiuqin Xu, Haitao Lv, Ling Sun

**Affiliations:** Department of Cardiology, Children’s Hospital of Soochow University, Suzhou, China

**Keywords:** hypertension, children, neutrophil-lymphocyte ratio, left ventricular hypertrophy, left ventricular diastolic function

## Abstract

**Aim:** Hypertension is associated with cardiac structural and functional changes, including left ventricular hypertrophy (LVH) and LV systolic dysfunction diastolic dysfunction. Neutrophil-to-lymphocyte ratio (NLR) is a novel inflammatory biomarker associated with cardiovascular diseases. The current study aimed to evaluate NLR in children with newly diagnosed essential hypertension and its relationship between blood pressure and cardiac changes.

**Methods and Subjects:** Sixty-five children with newly diagnosed essential hypertension and 54 healthy children were included. Clinical characteristics, blood cell counts, and biochemical parameters were collected. LVH was assessed by calculation of LV mass index (LVMI), and LV systolic function was evaluated by measuring LV ejection fraction and fractional shortening. LV diastolic function was primarily assessed with E/E′ ratio by Doppler and echocardiography.

**Results:** The hypertension children had significantly higher LVMI and E/E′ ratio than the controls, whereas there was no difference in LV systolic function between the two groups. The NLR was significantly higher in the hypertension group than the control group. Moreover, NLR was positively correlated with systolic blood pressure (SBP) and diastolic blood pressure (DBP) levels in the hypertension group. Additionally, a significantly positive correlation between NLR and E/E′ ratio was found in the hypertension group. However, NLR was not related to LVH and LV systolic function indicators in hypertension children.

**Conclusion:** NLR is elevated in hypertension children, and it is associated positively with office blood pressure levels. Moreover, NLR may help assess LV diastolic function in hypertension children.

## Background

Hypertension is the leading risk factor for cardiovascular disease and mortality in adults, with a prevalence of 31.1% worldwide (1). In parallel with the growing prevalence of childhood obesity, it is becoming an increasing problem among children over the last few decades, as a consequence of obese children who are at approximately a three-fold higher risk for hypertension than non-obese children (2). According to the American Heart Association, the prevalence of high blood pressure is 14.2% for US children (3), and the incidence of high blood pressure is 14.13–17.00% for children aged 7–17 years in China (4). Of note, a recent longitudinal study has demonstrated that blood pressure in childhood is the strongest independent predictor of future blood pressure in adulthood (5), emphasizing the importance of blood pressure management in childhood.

Growing evidence shows that hypertension results in target organ damage, even in prehypertension children (6). The increased left ventricular mass (LVM) and cardiac function abnormalities are the early change in target organ damage (7). Therefore, screening for rapid and straightforward indicators to reflect the target organ damage has become a useful strategy in managing childhood hypertension.

Over the last years, comprehensive data have demonstrated the pivotal role of low-grade inflammation in the pathogenesis of essential hypertension and target organ damage in both adults (8) and children (9). The white blood cells (WBCs) and their subtypes with platelets are the essential cells of inflammation. Therefore, blood cell parameters have attracted increasing attention in chronic inflammation disease. The neutrophil-to-lymphocyte ratio (NLR), lymphocyte-to-monocyte (LMR), and platelet-to-lymphocyte ratio (PLR) were proposed as the inexpensive, easily accessible, and widely available inflammatory markers. They have been shown to be related to cardiovascular diseases in adults, including atherosclerosis (10), heart failure (11), acute coronary syndromes (12), and hypertension (13). Moreover, Skrzypczyk et al. reported that NLR correlated with 24-h ambulatory mean arterial pressure levels in adolescents (14), which suggest that blood cell parameter may also be useful in the pediatrics population. To date, no studies have investigated the possible link between NLR and target organ damage in hypertension children. Therefore, our study aimed to evaluate blood cell count inflammatory markers in children with newly diagnosed essential hypertension and explore the possible link between NLR and cardiac structural and functional changes.

## Methods

### Ethics Committee Approval

The local Ethics Committee of Children’s Hospital of Soochow University approved the research project (ID: 2020CS076). All procedures performed involving human participants followed the Declaration of Helsinki, and informed consent was obtained from all participants and their parents included in the study.

### Study Group

In the current study, we retrospectively studied 65 children (48 boys, 17 girls) with newly diagnosed essential hypertension hospitalized in the cardiology department in the Children’s Hospital of Soochow University from January 2016 to December 2020, and 54 age and sex-matched healthy children were recruited from the community-based population as the control group.

Clinical parameters, including age, gender, and body mass index (BMI; kg/m^2^) were obtained in all analyzed children. Hypertension was defined as systolic and/or diastolic pressure ≥95th percentile for sex, age, and height according to the reference value of the Chinese Child Blood Pressure References Collaborative Group (15). Office blood pressure was measured by an automated oscillometric device (Datascope Accutor Plus) with the appropriate size cuff that had been validated for use in children (16). The appropriate cuff size (with bladder width of about 40–50% of the arm circumference and the bladder length of at least 80% of the arm circumference) was determined by measuring the mid-upper arm circumference. Blood pressure was measured in the non-dominant arm in triplicate at 3-min intervals after a 15 min rest in the sitting position with the arm and back supported. After excluding the first reading, the average of two subsequent readings was calculated for analysis.

To exclude secondary hypertension, a thorough medical history, physical examination, and auxiliary examination was carried out following the guideline of the American Academy of Pediatrics (17). In addition, based on medical history, physical examination, and determined high-sensitivity C-reactive protein (hsCRP) levels, children with active inflammation were excluded in the current study.

### Laboratory Assessment

Blood was obtained from an antecubital venous catheter after 10–12 h of night fasting. All specimens were EDTA-K2 anticoagulated and tested within 30 min of collection. The hematological parameters, including white blood cell (WBC), differential WBC counts (neutrophils, lymphocytes, and monocytes), and platelet count (Plt) were measured by an automated hematology analyzer. The neutrophil-to-lymphocyte ratio (NLR), lymphocyte-to-monocyte ratio (LMR), and platelet-to-lymphocyte ratio (PLR) were calculated.

Moreover, plasma glucose, triglycerides, total cholesterol, high-density lipoprotein cholesterol (HDL-c), low-density lipoprotein cholesterol (LDL-c), hsCRP, alanine aminotransferase (ALT), and creatinine were determined at the Department of Clinical Laboratory of the Children’s Hospital of Soochow University.

### Echocardiographic Assessment

All echocardiographic parameters were performed using commercially available ultrasound equipment iE33 (Phillips Healthcare, North Andover, Massachusetts, USA).

### Left Ventricular Geometry

The M-mode tracing was used to measure the end-diastolic interventricular septal wall thickness (IVSd), left ventricular end-diastolic diameter (LVIDd), left ventricular end-systolic diameter (LVIDs), and end-diastolic posterior wall thickness (PWTd). The left ventricular mass (LVM) was then calculated using the following formula: LVM = 0.8 × 1.04 × [(IVSd + LVIDd + PWTd)^3^ – LVIDd^3^] + 0.6, LVM index (LVMI) = LVM/height^2.7^, relative wall thickness (RWT) = (IVSd + PWTd)/LVIDd. LV hypertrophy (LVH) in children and adolescents is defined as the LVMI ≥ 95th percentile on sex-specific normative LVMI data published by Khoury et al. (18).

### Left Ventricular Systolic Function

LV systolic function was assessed by the LV ejection fraction (EF) and fractional shortening (FS) (19).

### Left Ventricular Diastolic Function

#### Pulsed Doppler Assessment

Mitral inflow velocities were acquired with pulsed wave Doppler. The velocities during the early transmitral flow (E) and inflow with atrial contraction (A) were measured, and the E/A ratio was calculated.

#### Tissue Doppler Imaging

Myocardial flow velocities were obtained in the apical four-chamber view. The peak early E′ and late A′ velocities were recorded, then the E′/A′ ratio and E/E′ ratio were calculated (20), and the left ventricular diastolic dysfunction was defined as E/E′ ratio > 14, according to the recommendations of the American Society of Echocardiography (21).

### Statistics

Statistical analyses were performed using SPSS 22.0 (SPSS Inc., Chicago, IL). Values were expressed as mean and SD. The Shapiro–Wilk-test was used to determine the normality of data. Means were compared using an independent *t*-test between hypertension and control groups. Categorical variables were compared using the chi-square test. Correlations between variables were evaluated using Pearson’s tests. Multivariate linear analyses were performed to estimate the association of potential confounding factors between the LV diastolic function indicators. A *P*-value < 0.05 was considered significant.

## Results

### Clinical Characteristics and Biochemical Parameter in Hypertension and Control Group

**Figure 1:**
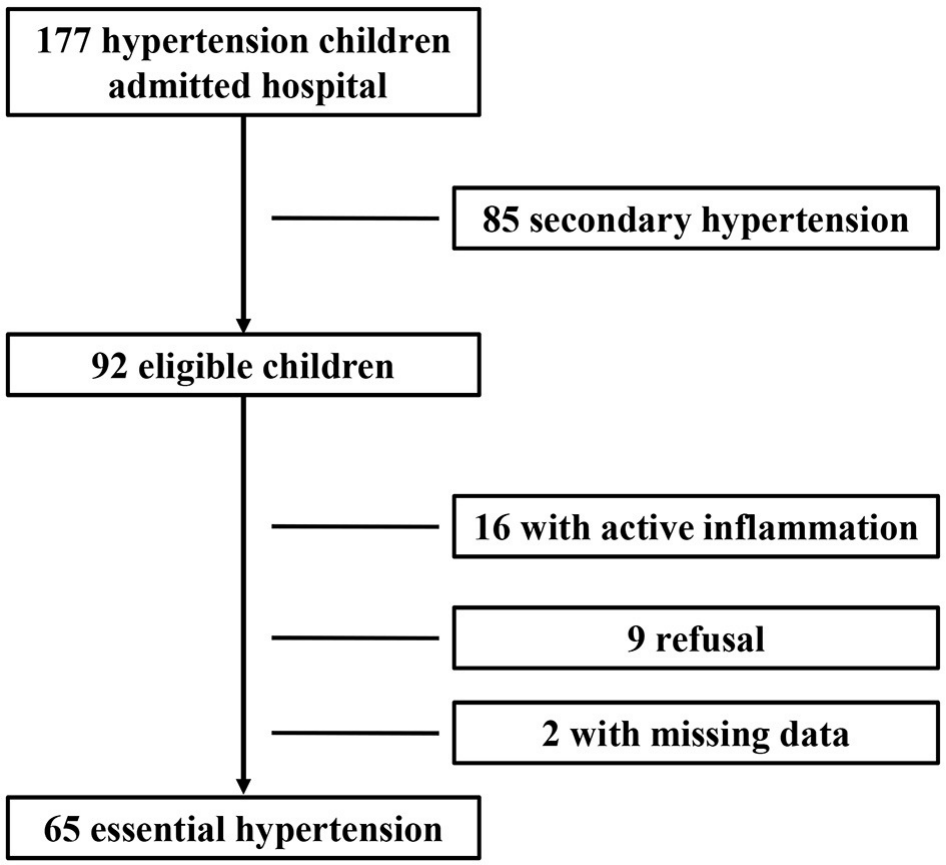
Participant selection process.

During the study period, 177 children with newly diagnosed hypertension were hospitalized. Among them, 85 cases with secondary hypertension and 16 cases with active inflammation and two cases with missing data were excluded; and nine cases refused to participate. Therefore, 65 children with newly diagnosed essential hypertension were evaluated, and the selection process is shown in Figure 1. The clinical characteristics and biochemical parameters of hypertension and control groups are shown in Table 1. There was no difference in terms of sex and age between the two groups. SBP, DBP, pulse pressure, and BMI were significantly higher in the hypertension group than in the control groups. Also, the serum uric acid, ALT, and hsCRP levels in children with hypertension were significantly higher than in the control group, respectively. However, there were no differences in lipids and glucose levels between the two groups (Table 1).

**Table 1:** Clinical characteristics and biochemical parameter in hypertension group and control group.

	**Control group**	**Hypertension group**	***P-*value**
**Clinical characteristics**			
Gender (M/F)	43/11	48/17	0.459
Age, years	12.33 ± 2.3	12.37 ± 2.34	0.937
BMI (kg/m^2^)	108.52 ± 9.47	141.08 ± 11.95	0.003
SBP (mmHg)	67.75 ± 7.63	85.15 ± 11.66	<0.001
DBP (mmHg)	35.87 ± 15.11	55.05 ± 13.68	<0.001
PP (mmHg)	18.77 ± 2.06	26.62 ± 14.62	<0.001
**Biochemical variables**			
Uric acid, μmol/L	328.99 ± 83.26	407.11 ± 111.07	<0.001
ALT, μmol/L	15.24 ± 8.76	31.49 ± 41.66	0.004
Creatinine, μmol/L	69.28 ± 17.34	66.15 ± 15.47	0.311
TG, mmol/L	1.14 ± 0.50	1.14 ± 0.49	0.950
TC, mmol/L	3.64 ± 1.12	3.87 ± 0.98	0.296
HDL-c, mmol/L	1.22 ± 0.27	1.26 ± 0.41	0.654
LDL-c, mmol/L	1.94 ± 0.73	2.16 ± 0.97	0.348
Glucose, mmol/L	4.14 ± 1.45	4.27 ± 1.55	0.612
hsCRP, mg/dl	0.32 ± 0.42	2.22 ± 5.03	0.004

*SBP, systolic blood pressure; DBP, diastolic blood pressure; PP, pulse pressure; TG, triglyceride; TC, total cholesterol; HDL-c, high density lipoprotein cholesterol; LDL-c, low density lipoprotein cholesterol; ALT, alanine aminotransferase; hsCRP, high-sensitivity C-reactive protein*.

### Echocardiography Parameters in Hypertension and Control Group

The LVM, LVMI, and RWT were higher in the hypertension group compared with the control group, and 8 of 65 children in the hypertension group had LVH (12.31%). Besides, the E/E′ ratio was higher in the hypertension group in comparison with the control group, LV diastolic dysfunction was found in 1 of the 65 hypertensive subjects (1.54%) (Table 2). However, there was no difference in LV ejection fraction and FS between the two groups.

**Table 2:** Echocardiography parameters in the hypertension group and control group.

	**Control group**	**Hypertension group**	***P-*value**
LVM, g	105.61 ± 29.6	139.32 ± 49.1	<0.001
LVMI, g/m^2.7^	29.42 ± 6.56	37.71 ± 10.67	<0.001
RWT (%)	0.30 ± 0.04	0.35 ± 0.06	<0.001
LV hypertrophy, *n* (%)	/	8 (12.31%)	/
E′, cm/s	12.66 ± 1.84	12.62 ± 1.77	0.898
E′/A′ ratio	2.05 ± 0.56	2.25 ± 1.13	0.295
E/A ratio	2.17 ± 0.59	1.92 ± 0.59	0.033
E/E′ ratio	7.77 ± 1.63	8.53 ± 1.79	0.038
Diastolic dysfunction, *n* (%)	/	1 (1.54%)	/
LVEF (%)	72.52 ± 3.85	71.6 ± 6.23	0.389
FS (%)	42.42 ± 4.71	43 ± 6.21	0.606

*LVM, left ventricular mass; LVMI, left ventricular mass index; RWT, relative wall thickness; LVEF, left ventricular ejection fraction; FS, fractional shortening*.

### Blood Cell Counts in Hypertension and Control Group

The WBC count and neutrophil counts were significantly higher in hypertension children than those in the control group, whereas lymphocyte, monocytes, and platelet counts were similar between the two groups (Table 3). Moreover, NLR is higher in the hypertension group than the control group. However, there was no difference in PLR and LMR between hypertension and control groups (Table 3).

**Table 3:** Blood cell count inflammatory markers in hypertension and control group.

	**Control group**	**Hypertension group**	***P-*value**
WBC (10^9^/L)	6.70 ± 1.71	7.65 ± 2.27	0.017
N (10^9^/L)	3.77 ± 1.27	4.62 ± 1.72	0.003
L (10^9^/L)	2.42 ± 0.76	2.43 ± 0.91	0.931
M (10^9^/L)	0.41 ± 0.13	0.46 ± 0.16	0.089
Plt (10^9^/L)	280.66 ± 76.35	287.48 ± 77.57	0.110
NLR	1.68 ± 0.75	2.18 ± 1.12	0.005
PLR	122.46 ± 36.71	132.08 ± 57.77	0.287
LMR	6.19 ± 1.74	6.41 ± 7.2	0.832

*WBC, white blood cell count; N, neutrophils count; L, lymphocytes count; M, monocytes count; Plt, platelet count; NLR, neutrophil-to-lymphocyte ratio; PLR, platelet-to-lymphocyte ratio; LMR, lymphocyte-to-monocyte ratio*.

### Correlation Between Blood-Cell Count Inflammatory Markers and Office Blood Pressure With Body Mass Index in Hypertension Children

**Figure 2:**
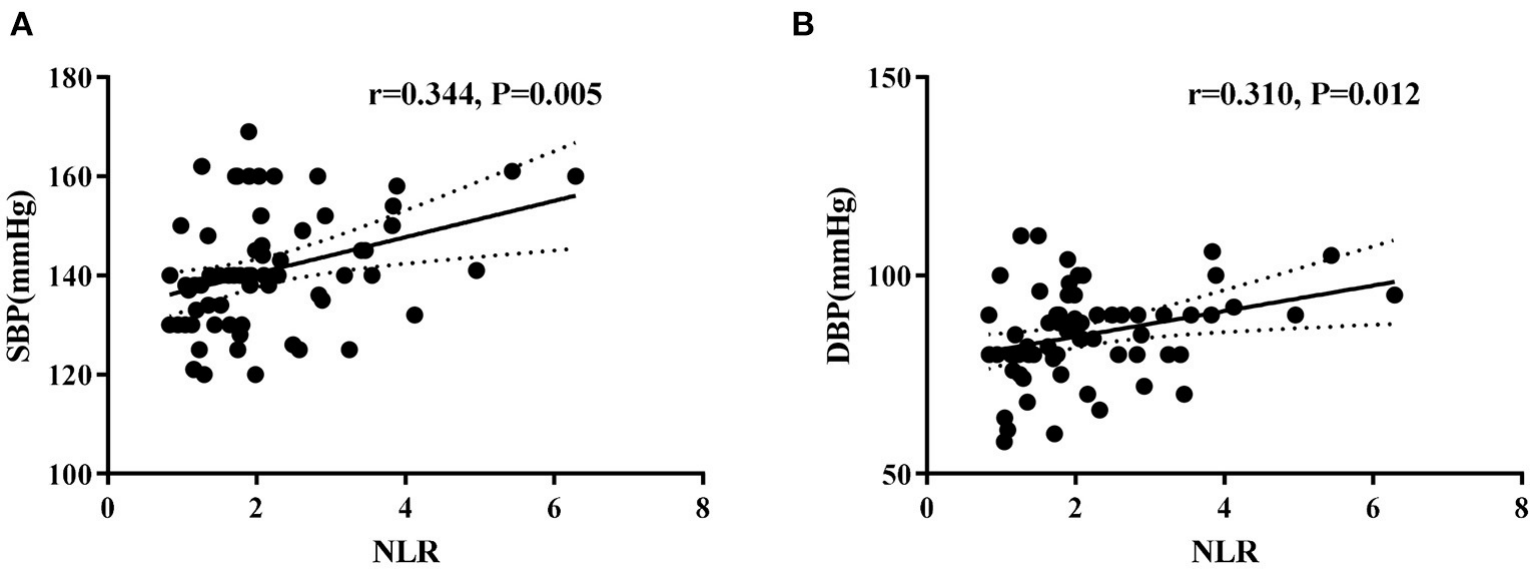
Correlations between neutrophil-to-lymphocyte ratio (NLR) and systolic blood pressure **(A)** and diastolic blood pressure **(B)** levels in hypertension children.

In the hypertension group, univariate correlation analysis determined a significant positive correlation between NLR with office SBP (*r* = 0.344, *P* = 0.005) and DBP (*r* = 0.310, *P* = 0.012) levels (Figure 2). However, NLR was not associated with BMI levels. Moreover, WBC counts, neutrophil counts, lymphocyte counts, monocytes counts, platelet counts, PLR, and LMR were not correlated with office blood pressure or BMI levels (Supplementary Table 1).

### Correlation Between Blood-Cell Count Inflammatory Markers and Left Ventricular Diastolic Function With Hypertrophy in Hypertension Children

**Figure 3:**
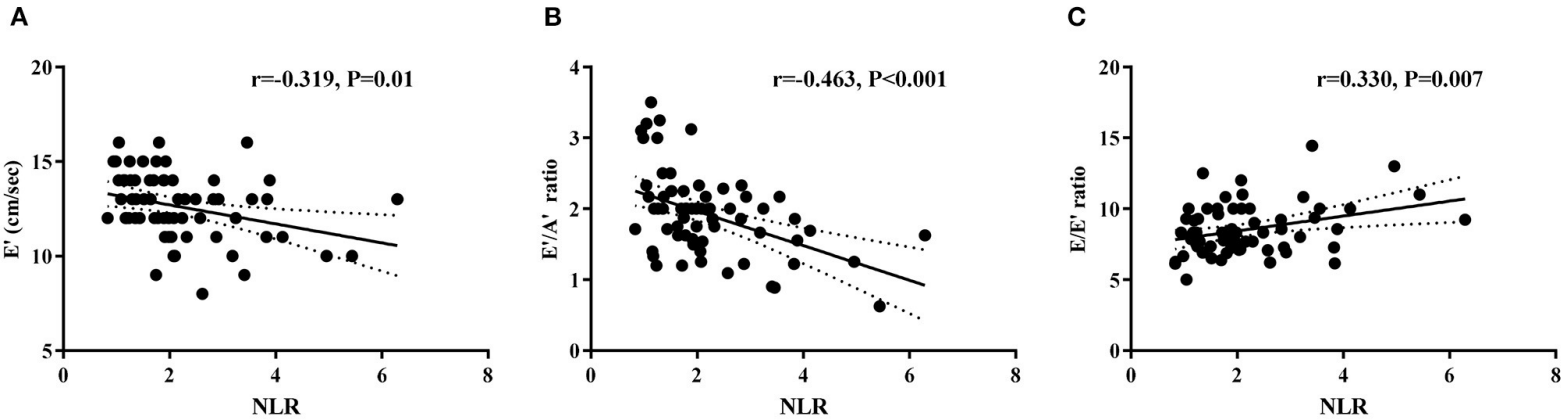
Correlations between NLR and left ventricular diastolic function parameters E′ **(A)**, E′/A′ ratio **(B)**, and E/E′ ratio **(C)** in hypertension children.

NLR was negatively correlated with E′ (*r* = −0.319, *P* = 0.010) and the E′/A′ ratio (*r* = −0.463, *P* < 0.001), and positively correlated with the E/E′ ratio (*r* = 0.330, *P* = 0.007) in the hypertension group (Figure 3). Neutrophil counts were also found to correlate negatively with E′ (*r* = −0.427, *P* = 0.001) and the E′/A′ ratio (*r* = −0.318, *P* = 0.036) in the hypertension group. Furthermore, after adjusting age, gender, and BMI, the multivariate regression analyses still detected the significant associations between NLR and E/E′ (β = 0.593, *P* = 0.003, 95% CI: 0.209–0.978). However, there was no correlation between WBC, lymphocyte, monocytes, platelet counts, and BMI with diastolic function parameters (Supplementary Table 2).

On the other hand, these blood cell count inflammatory markers were not associated with left ventricular hypertrophy and systolic function parameters (Supplementary Table 3). However, BMI was positively correlated with LVMI in hypertension children (*r* = 0.588, *P* < 0.001).

## Discussion

The incidence of hypertension among children is overgrowing in recent years, and high blood pressure can induce cardiac structural and functional target organ damage (22). Our study demonstrated the elevation of LVMI and the reduction of diastolic function in newly diagnosed essential hypertension children. Moreover, we found elevated NLR in the hypertension group, and it is positively correlated with office blood pressure levels, which might imply the possible link between the inflammation and elevation of blood pressure in the hypertension children. Interestingly, NLR was positively correlated with left ventricular diastolic parameter E/E′ ratio in hypertension children, which suggests that NLR may serve as a useful indicator to reflect diastolic dysfunction in hypertension children. To our knowledge, this is the first study that analyzes the change in NLR and its relationship between LV diastolic function in newly diagnosed essential hypertension children.

It is well-established that LVH is an independent risk factor for cardiovascular morbidity and mortality in adulthood (23, 24). Previous studies have proved that LVH was common in hypertensive children (25). However, there is limited data available in the Chinese pediatric population. In this study, we found that LVM and LVMI were both higher in hypertension children, and among 65 hypertension adolescents, 12.31% (8/65) had LVH. Similarly, Litwin et al. demonstrated that 10.3% of 44 hypertension children had some form of LV hypertrophy in the USA population (26), and Falkner reported among 35 African-American adolescents, 19% of them had LV hypertrophy (27). A cross-sectional study of 101 primary hypertension children also reported that 34% of them had LV hypertrophy (28). All these studies demonstrated that LVH was common in hypertensive children. However, the prevalence of LVH varies among these studies, which may be explained by the differences in ethnic and hypertension grades of these participants. Interestingly, BMI was positively correlated with LVMI in hypertension children, and these findings are consistent with the direct positive correlation between BMI and LVH among obese adults in a systematic review (29), which indicates the possible additive and/or interactive effects of obesity and blood pressure on LVH (30).

Several studies have shown that NLR is elevated and related to poor clinical outcomes of cardiovascular disease in adults, including acute coronary syndrome, atherosclerosis, and heart failure (31–33). However, few studies have looked at NLR levels in children with newly diagnosed hypertension (14, 34). In the current study, we found that the neutrophil counts and NLR were significantly higher in hypertension children than in healthy children, and the elevated NLR may reflect the upregulation of overall inflammatory and stress status in these children. Likewise, Derya et al. found that NLR is increased in newly diagnosed hypertension adults and associated positively with low-grade inflammation indicator C-reactive protein levels (13), which is consistent with our findings.

Furthermore, we demonstrated that NLR positively correlated with both office SBP and DBP levels in hypertension children. Similarly, Cimen et al. reported that NLR correlated with blood pressure levels in adults (35). The increase in NLR may reflect the activity of two different immune pathways in the process of blood pressure regulation. On the one hand, neutrophils secrete many cytokines that trigger and amplify inflammatory reactions (36), and activated neutrophils’ release of various proteolytic enzymes that promote endothelial damage and tissue destruction (37, 38). Also, neutrophils can lead to the release of reactive oxygen species (39), and ROS-induced oxidative stress has been shown to cause vasoconstriction (40) with sodium and water retention in the kidney (41). On the other hand, lymphocytes are the primary cells in the regulatory pathway of the immune system, and T lymphocytes cells have been shown to play a crucial role in the BP elevation caused by angiotensin II response to sodium and volume challenges (42). Therefore, NLR gives more information than either of the above parameters in hypertension, which indicates that inflammation may play an essential role in the development of hypertension.

The possible links between NLR and cardiac damages in these hypertension children were also demonstrated in this study. LVH and LV diastolic dysfunction are both the early complication of hypertension (43); and eight children had LVH, and one child had diastolic dysfunction among these hypertension participants. As known, the E/E′ ratio seems to be the most reliable parameter to evaluate LV diastolic function in patients with heart disease, and the increase in E/E′ ratio reflects LV diastolic dysfunction (44). Interestingly, we found that NLR positively correlated with E/E′ ratio among hypertension children; after adjusting for the potential confounding covariates, including BMI, gender and age, NLR still correlated with E/E′ ratio. To our best knowledge, this is the first study to report the correlation between NLR and diastolic function in hypertension children. Since no correlation between blood cell count inflammatory markers and LVH parameters was demonstrated in this study, which suggests that the LV diastolic dysfunction in these hypertensive children is probably due to systematic inflammation (45) rather than left ventricular hypertrophy. These results indicate that NLR may serve as a helpful marker to evaluate the LV diastolic function in hypertension children.

There are several limitations to this study. First, this is a single-center retrospective study, and the sample size is relatively small, along with the large burden of multiple analyses, which increases possible type I error. Second, the gender of hypertension children is unbalanced in this study. Recently, no difference in the global prevalence of hypertension in children was found in a meta-analysis (46). However, the prevalence of hypertension among school-age children was higher in boys than in girls (16.1 vs. 12.9%), according to an updated Report on Cardiovascular Health and Diseases in China (47). Therefore, the gender-specific prevalence of child hypertension might vary among different regions. Moreover, these hypertension children were recruited from a hospital-based population, which may also increase the risk of selective bias and resulting in gender discrepancies. Third, since this is an observational study, we cannot make any causal inferences. Fourth, due to the physiological characteristics in the blood cell counts of children under 5 years of age (48), the results cannot be extrapolated to this population.

In conclusion, we demonstrated that NLR is elevated in hypertension children, and it is associated positively with office blood pressure levels and LV diastolic dysfunction parameters. Our results indicate that inflammation may play a crucial role in the development of hypertension, and the higher NLR may indicate the increased risk for the development of hypertension in children. Moreover, NLR can serve as a useful marker to reflect left ventricular diastolic dysfunction in pediatric patients with primary hypertension.

## Data Availability Statement

The raw data supporting the conclusions of this article will be made available by the authors, without undue reservation.

## Ethics Statement

The studies involving human participants were reviewed and approved by The Ethics Committee of Children’s Hospital of Soochow University. Written informed consent to participate in this study was provided by the participants’ legal guardian/next of kin.

## Author Contributions

MH, LC, YD, and LS conceived and designed the study and wrote the paper. MH, LC, YD, YC, BW, JS, WZ, JH, QX, HL, and LS performed the study. YC, BW, JS, WZ, JH, QX, and HL reviewed and edited the manuscript. All authors read and approved the manuscript.

## Conflict of Interest

The authors declare that the research was conducted in the absence of any commercial or financial relationships that could be construed as a potential conflict of interest.
