# Supplementary material for: Neutrophil to Lymphocyte Ratio Is Increased and Associated With Left Ventricular Diastolic Function in Newly Diagnosed Essential Hypertension Children
Source: Front Pediatr. 2021 May 19;9:576005. doi: 10.3389/fped.2021.576005 (PMC8169980; doi:10.3389/fped.2021.576005)
Supplement: Supplementary file 1 [file Data_Sheet_1.docx]

**Supplemental Table 1. Correlations between blood-cell count inflammatory markersand office blood pressure levels in hypertension children**

|  | SBP | | DBP | | BMI | |
| --- | --- | --- | --- | --- | --- | --- |
|  | r | *P* | r | *P* | r | *P* |
| WBC | 0.211 | 0.099 | 0.034 | 0.793 | -0.128 | 0.324 |
| N | 0.319 | 0.071 | 0.153 | 0.236 | -0.105 | 0.419 |
| L | -0.068 | 0.602 | -0.192 | 0.135 | -0.082 | 0.531 |
| M | 0.124 | 0.337 | -0.108 | 0.401 | -0.073 | 0.574 |
| Plt | 0.077 | 0.554 | 0.055 | 0.673 | 0.072 | 0.583 |
| NLR | 0.344 | 0.005* | 0.310 | 0.012* | -0.129 | 0.308 |
| PLR | 0.163 | 0.205 | 0.258 | 0.043 | 0.155 | 0.232 |
| LMR | -0.068 | 0.600 | 0.017 | 0.893 | 0.028 | 0.830 |

* *P* <0.05

SBP, systolic blood pressure; DBP, Diastolic blood pressure; WBC, white blood cell count; N, neutrophils count; L, lymphocytes count; M, monocytes count; Plt, platelet count; NLR, neutrophil-to-lymphocyte ratio; PLR, platelet-to-lymphocyte ratio; LMR,lymphocyte-to- monocyte ratio.

**Supplemental Table 2. Correlations between blood cell counts parameters and left ventricular diastolic function parameters in hypertension children.**

|  | E’ | | E/A | | E’/A’ | | E/E’ | |
| --- | --- | --- | --- | --- | --- | --- | --- | --- |
|  | r | *P* | r | *P* | r | *P* | r | *P* |
| WBC | -0.339 | 0.007 | -0.252 | 0.099 | -0.074 | 0.569 | 0.096 | 0.457 |
| N | -0.427 | 0.001* | -0.318 | 0.036* | -0.270 | 0.034 | 0.195 | 0.129 |
| L | 0.018 | 0.889 | -0.051 | 0.743 | 0.313 | 0.013 | -0.144 | 0.263 |
| M | -0.164 | 0.201 | 0.044 | 0.775 | 0.126 | 0.328 | 0.062 | 0.632 |
| Plt | -0.028 | 0.829 | -0.021 | 0.891 | -0.144 | 0.264 | 0.005 | 0.966 |
| NLR | -0.319 | 0.010* | -0.194 | 0.206 | -0.463 | <0.001* | 0.330 | 0.007* |
| PLR | -0.052 | 0.688 | 0.075 | 0.628 | -0.322 | 0.011 | 0.132 | 0.308 |
| LMR | -0.142 | 0.273 | -0.134 | 0.386 | -0.002 | 0.989 | -0.090 | 0.488 |

* *P* <0.05

WBC, white blood cell count; N, neutrophils count; L, lymphocytes count; M, monocytes count; Plt, platelet count; NLR, neutrophil-to-lymphocyte ratio; PLR, platelet-to-lymphocyte ratio; LMR,lymphocyte-to- monocyte ratio.

**Supplemental Table 3. Correlations between blood-cell count inflammatory markersand left ventricular hypertrophy and systolic function parameters in hypertension children.**

|  | FS | | EF | | LVM | | LVMI | | RWT | |
| --- | --- | --- | --- | --- | --- | --- | --- | --- | --- | --- |
|  | r | P | r | P | r | P | r | P | r | P |
| WBC | -0.101 | 0.496 | -0.171 | 0.246 | -0.166 | 0.255 | -0.145 | 0.319 | -0.014 | 0.926 |
| N | -0.051 | 0.732 | -0.180 | 0.220 | -0.143 | 0.328 | -0.180 | 0.215 | -0.026 | 0.861 |
| L | -0.153 | 0.298 | -0.116 | 0.434 | -0.156 | 0.285 | -0.054 | 0.713 | 0.004 | 0.980 |
| M | 0.054 | 0.715 | 0.052 | 0.723 | -0.016 | 0.915 | 0.058 | 0.693 | 0.060 | 0.681 |
| Plt | 0.193 | 0.189 | -0.002 | 0.991 | -0.198 | 0.174 | -0.075 | 0.611 | -0.150 | 0.303 |
| NLR | -0.136 | 0.357 | -0.153 | 0.300 | -0.179 | 0.220 | -0.172 | 0.238 | -0.083 | 0.570 |
| PLR | 0.171 | 0.244 | 0.088 | 0.554 | -0.083 | 0.569 | 0.005 | 0.975 | -0.168 | 0.247 |
| LMR | -0.157 | 0.287 | -0.118 | 0.425 | 0.019 | 0.895 | 0.001 | 0.995 | 0.061 | 0.677 |
| BMI | -0.008 | 0.956 | 0.133 | 0.374 | 0.038 | 0.797 | 0.588 | <0.001* | -0.014 | 0.924 |

* *P* <0.05

WBC, white blood cell count; N, neutrophils count; L, lymphocytes count;

M, monocytes count; Plt, platelet count; NLR, neutrophil-to-lymphocyte ratio; PLR, platelet-to-lymphocyte ratio; LMR,lymphocyte-to- monocyte ratio. FS, Fractional Shortening; EF, Ejection fraction; LVM, left ventricular mass; LVMI, left ventricular mass index; RWT, Relative wall thickness.
